# Identification of diagnostic discrepancies as a quality assurance measure in emergency medicine – a validation study

**DOI:** 10.1186/s13049-026-01572-x

**Published:** 2026-02-11

**Authors:** Thimo Marcin, Nadine Werthmüller, Fabian Kölbener, Martin Müller, Laura Zwaan, Stefanie C. Hautz, Alexander Schuster, Aristomenis K. Exadaktylos, Wolf E. Hautz

**Affiliations:** 1 Department of Emergency Medicine, Inselspital University Hospital Bern, University of Bern, Freiburgstrasse, Bern, 3010 Switzerland; 2 Faculty of Medicine, University of Bern, Bern, Switzerland; 3 IMERR, Institute of Medical Education Research Rotterdam, Erasmus MC, Rotterdam, The Netherlands; 4 MVZ Jung-Stilling, Siegen, Germany

**Keywords:** Diagnostic errors, Misdiagnosis, Emergency medicine

## Abstract

**Background:**

Diagnostic errors are a major care health concern but remain difficult to study because their identification often requires resource-intensive chart reviews. We aimed to validate a previously proposed automated method for detecting discrepancies between an initial and a later, more definitive diagnosis as a screening tool for potential diagnostic errors in a large, prospective cohort of emergency department (ED) patients.

**Methods:**

This secondary analysis included 1,204 patients enrolled in the DDxBRO randomized trial, which evaluated the effect of a diagnostic decision support tool on diagnostic quality in four Swiss emergency departments. For each patient, the ED diagnosis was extracted from the ED discharge letter, and the follow-up diagnosis at 14 days was obtained from hospital discharge letters, or general practitioner notes. All diagnoses were coded using ICD-10 and manually classified for discrepancies by two blinded ED physicians according to a predefined scheme. The automated method calculated the “similarity” between ICD-10 codes for ED and follow-up diagnoses. Discriminative performance of this method to distinguish between cases with and without diagnostic error was evaluated using receiver operating characteristic (ROC) curves, and sensitivity, specificity, and predictive values were assessed across multiple cutoffs.

**Results:**

The automated method showed high and consistent discriminative performance across all algorithms tested, with areas under the ROC curve (AUCs) ranging from 0.94 to 0.95. Using the most sensitive cutoff in the simplest algorithm, all true discrepancies were detected, but 162 cases (15%) were incorrectly flagged as discrepant.

**Conclusion:**

The automated method demonstrated high accuracy and shows promise as a practical screening tool to prioritize cases for resource-intensive chart review.

**Trial registration:**

NCT05346523.

**Supplementary Information:**

The online version contains supplementary material available at 10.1186/s13049-026-01572-x.

## Introduction

### Background

Diagnostic errors are a major healthcare problem, affecting 5–15% of patients seeking medical care and contributing substantially to patient harm [1–3]. Such errors are associated with higher mortality rates compared to other types of medical errors [4] and represent the leading cause of costly malpractice claims [5].

Diagnostic errors are commonly defined as delayed, wrong or missed diagnoses [1]. This definition implies a substantial discrepancy between the diagnostic label assigned during an index medical visit and a somehow more precise, accurate or otherwise better diagnosis later on [6, 7].

Emergency Medicine (EM) is a field that is particularly prone to diagnostic error for several reasons. Many diagnoses are first established in the emergency department (ED), often without an ongoing, long-standing patient-physician relationship to provide diagnostic context. Physicians in the EM frequently work under significant time pressure and high patient volumes, and since EM is a 24/7 specialty, both clinicians and patients are prone to fatigue. These factors collectively heighten the risk of errors throughout the diagnostic process [8–10].

### Importance

A recent, though heavily debated, review estimated that as many as 350.000 patients in the United States die or suffer permanent disability each year due to diagnostic errors in EM [9]. This review has been criticized because its most important estimates were derived from only three primary studies [11, 12]. This lack of research on diagnostic quality in emergency medicine is particularly surprising, because the landmark report “Improving diagnosis in medicine” was published nearly a decade ago by the Institute of Medicine (now National Academy of Medicine) [1]. Since then, only few researchers pursued its call to investigate diagnostic quality in EM.

Reasons for this lack of research into diagnostic quality in EM may be the complexity of measuring diagnostic errors and the large effort required to conduct such studies. Identifying diagnostic errors often require full case review by experts [13]. However, following up on the diagnosis of 100.000 or more patients seen annually in any given ED may simply not be feasible.

One strategy to reduce the number of cases requiring manual chart review and increase the efficiency of follow-up is the use of trigger tools. A trigger is an easy to measure event in widely available, preferably routine data that is associated with the event or outcome of interest. Several triggers have been proposed to identify potential diagnostic errors, such as unplanned medical revisits within 3, 7 or 14 days, intensive care unit admission from the general ward after admission from the ED, or death within a given timeframe [14–16]. However, the sensitivity and specificity of these triggers are largely unknown in relation to diagnostic error. To advance research into diagnostic quality in EM, it would be preferable to directly identify outcomes relevant to diagnostic accuracy, such as discrepancies between the diagnostic label assigned at an index ED visit and a subsequently established diagnosis. We have previously demonstrated that diagnostic labelling errors are associated with longer hospital stay and increased mortality [17]. However, this determination currently requires time-consuming manual chart review by expert clinicians to distinguish true diagnostic errors from changes due to evolving clinical information or disease progression. Automating the identification of diagnostic discrepancies could provide an efficient, scalable trigger tool to screen for potential errors, thereby reducing the burden of full manual review.

### Goals of this investigation

We previously introduced an automated method to quantify the similarity between diagnostic labels [18]. This method leverages the hierarchical structure of the International Classification of Diseases (ICD), which organizes diagnoses as leaves on a branching tree. Closely related diagnoses share a small branch (e.g., subtypes of hypertension), while more distant diagnoses are separated by larger branches (e.g., from “diseases of the circulatory system” to “respiratory disorders”). The distance between two diagnostic labels is defined as the number of steps required to traverse from one leaf to another, providing a metric of diagnostic similarity or discrepancy. In a smaller prior study, we demonstrated that this method corresponds well with expert physicians’ judgement of whether two given diagnoses are similar or discrepant [18]. The purpose of the current study is to validate this approach in a large, multicenter cohort of ED patients [14] and evaluate its potential as automated trigger tool to identify diagnostic labelling in errors in EM using routinely available data.

## Methods

### Design

This study is a secondary analysis of data collected during the DDxBRO trial, a randomized controlled trial conducted in four Swiss emergency departments to evaluate the effect of a computerized diagnostic decision support tool on diagnostic quality [14, 19]. In brief, adults presenting to the ED with abdominal pain, fever, syncope, or other non-specific symptoms such as generally degraded condition or generalized weakness were diagnosed either with or without the aid of the differential diagnosis tool. Participants were followed for 14 days, and both their ED discharge diagnosis and any subsequent diagnosis for the same presenting complaint were recorded.

The primary endpoint of the original trial was a composite diagnostic quality risk score, including diagnostic labeling error within 14 days. The study was approved by the relevant local ethics committees and the Swiss national regulatory authority for medicinal products and is registered on ClinicalTrials.gov (NCT05346523).

### Participants and follow-up

The DDxBRO trial enrolled 1204 emergency patients (591 female, 613 male) aged 16 years or older. All participants provided written informed consent prior to study enrollment. For all patients, ED discharge diagnoses and current diagnoses at follow-up were collected. Follow-up data were obtained by trained study nurses, blinded to the study intervention, through review of hospital electronic health records, direct phone contact with patients or their general practitioners. Diagnoses were collected in narrative form from these sources, no diagnoses were collected from patients. All diagnoses were then coded using ICD-10 by two trained physicians. Patients were classified as “lost to follow-up” if they could not be reached after three attempts on three different days and if no additional health status information was available from their general practitioner or the hospital’s electronic health record.

## Diagnostic labelling errors

### Reference standard

Two experienced ED physicians (FK, NW) who were blinded and not otherwise involved in the trial assessed whether a diagnostic labelling error occurred according to predefined classification schemes (Supplemental Digital Content 1) previously published [17]. Cases with no follow-up medical care documented were assumed to have no change in diagnosis. The handling of diagnoses from ICD chapter R is detailed in the supplemental digital content.

To ensure consistency, both raters independently reviewed the first 40 cases, which were then discussed with a senior emergency physician (WEH) with expertise in diagnostic labeling errors. Inter-rater agreement was assessed on an additional 100 cases, yielding a moderate Cohen’s kappa of 0.4. Discrepancies in these initial 140 cases were resolved through discussion, and the remaining cases were classified individually by either rater. This expert manual classification of diagnosis pairs served as the gold standard for validating the automated diagnostic discrepancy metric used in this study.

### Index test

To complement manual classification, we applied our previously described automated ICD-based diagnostic discrepancy metric to each pair of diagnoses using the free software available at https://right-icd.ch/. The software offers four different algorithms to quantify the distance (or similarity) between two diagnoses in the ICD-10 hierarchy. The first is an unweighted path length, which counts the number of steps along the shortest path between two diagnoses. The second assigns different weights to edges in the ICD hierarchy to account for clinical similarity, giving higher weight to transitions between ICD chapters than within a single chapter. The third (Wu–Palmer) uses the position of two diagnoses relative to their most specific common ancestor in the hierarchy, while the fourth (Li et al.) combines path length and ancestor depth in a nonlinear function to better approximate human similarity ratings as described elsewhere [18].

### Statistical analysis

All analyses were performed using R version 4.2.2. Receiver operating characteristic (ROC) curves were generated for each algorithm, using the manually rated classification of diagnostic discrepancies (true/false) as the reference standard. The area under the curve (AUC) was calculated with 95% confidence intervals based on DeLong’s method, implemented in the pROC package. For the simplest step-count algorithm, we quantified sensitivity, specificity, and positive and negative predictive values across a range of cutoffs to evaluate discriminative performance. Optimal cutoffs, defined as the number of ICD-10 hierarchical steps that maximized sensitivity, specificity, or both, were identified using Youden’s Index and the OptimalCutpoints package.

## Results

A total of 1′204 patients at four EDs were enrolled and included in the intention-to-treat analysis of the DDxBRO main trial and thus in the following analyses. Patient characteristics have been described in detail elsewhere [19]. In brief, median age was 53 (interquartile range 34–69) and 51% were female patients. The majority (63%) of the patients presented with abdominal pain. Correspondingly, the primary ED diagnosis was most often a disease of the digestive system (ICD Chapter K00—K95 (Table 1). Expert human raters identified a diagnostic discrepancy between the primary ED diagnosis and follow-up diagnosis in 101 cases (8.4%). Overall, 303 patients were discharged from the ED without subsequent hospitalization or documented follow-up care with a general practitioner or clinic. All cause mortality during 14 day follow-up was 5 out of 1204 patients (0.42%). Of note, only one of these patients had a change in diagnosis recorded, unrelated to the cause of death.

**Table 1:** Patient characteristics

	**All Cases**	**Cases without a diagnostic discrepancy**	**Cases with a diagnostic discrepancy**
	**N = 1 204**	**N = 1 095**	**N = 101**
Age [years]	53 (34–69)	52 (33–68)	57 (36–75)
Female sex	613 (51%)	546 (50%)	63 (62%)
Chief complaint at ED admission			
Fever	173 (14%)	151 (14%)	20 (20%)
Abdominal Pain	754 (63%)	688 (63%)	63 (62%)
Syncope	156 (13%)	146 (13%)	8 (7·9%)
Non-Specific Complaint	121 (10%)	110 (10%)	10 (9·9%)
Charlson Comorbidity Index at ED admission			
0	502 (42%)	463 (42%)	37 (37%)
1–2	328 (27%)	305 (28%)	23 (23%)
3–4	230 (19%)	202 (18%)	24 (24%)
> = 5	144 (12%)	125 (11%)	17 (17%)
ICD10 Chapter of primary ED discharge diagnosis			
A00—B99 Certain infectious and parasitic diseases	155 (13%)	140 (13%)	14 (14%)
C00—D49 Neoplasms	18 (1·5%)	16 (1·5%)	2 (2·0%)
D50—D89 Diseases of the blood and blood-forming organs and certain disorders involving the immune mechanism	4 (0·3%)	4 (0·4%)	0 (0%)
E00—E89 Endocrine, nutritional and metabolic diseases	6 (0·5%)	6 (0·5%)	0 (0%)
F01—F99 Mental, Behavioral and Neurodevelopmental disorders	5 (0·4%)	5 (0·5%)	0 (0%)
G00—G99 Diseases of the nervous system	11 (0·9%)	11 (1·0%)	0 (0%)
H00—H59 Diseases of the eye and adnexa	1 (< 0·1%)	1 (< 0·1%)	0 (0%)
H60—H95 Diseases of the ear and mastoid process	11 (0·9%)	11 (1·0%)	0 (0%)
I00—I99 Diseases of the circulatory system	61 (5·1%)	57 (5·2%)	3 (3·0%)
J00—J99 Diseases of the respiratory system	82 (6·8%)	73 (6·7%)	7 (6·9%)
K00—K95 Diseases of the digestive system	337 (28%)	303 (28%)	32 (32%)
L00—L99 Diseases of the skin and subcutaneous tissue	4 (0·3%)	4 (0·4%)	0 (0%)
M00—M99 Diseases of the musculoskeletal system and connective tissue	26 (2·2%)	25 (2·3%)	1 (1·0%)
N00—N99 Diseases of the genitourinary system	142 (12%)	132 (12%)	10 (9·9%)
R00—R99 Symptoms, signs and abnormal clinical and laboratory findings, not elsewhere classified	284 (24%)	258 (24%)	26 (26%)
S00—T88 Injury, poisoning and certain other consequences of external causes	18 (1·5%)	15 (1·4%)	2 (2·0%)
U00—U49 Provisional assignment of new diseases of uncertain etiology or emergency use	36 (3·0%)	33 (3·0%)	3 (3·0%)
V00—Y99 External causes of morbidity	2 (0·2%)	1 (< 0·1%)	1 (1·0%)
Number of differential diagnoses besides primary ED discharge diagnosis			
0	945 (79%)	868 (79%)	71 (70%)
1	129 (11%)	110 (10%)	19 (19%)
2	103 (8·6%)	93 (8·5%)	9 (8·9%)
≥ 3	26 (2·2%)	24 (2·2%)	2 (2·0%)
Ratings of diagnostic discrepancy between primary ED and follow-up diagnosis			
Identical	932 (78%)	932 (85%)	0 (0%)
Precision	98 (8·2%)	98 (8·9%)	0 (0%)
Complication	65 (5·4%)	65 (5·9%)	0 (0%)
Diagnostically different	89 (7·4%)	0 (0%)	89 (88%)
Hierarchically different	12 (1·0%)	0 (0%)	12 (12%)

Median (Interquartile Range) for continuous data; n (%) for categorical data, *ED *Emergency Department

**Fig. 1:**
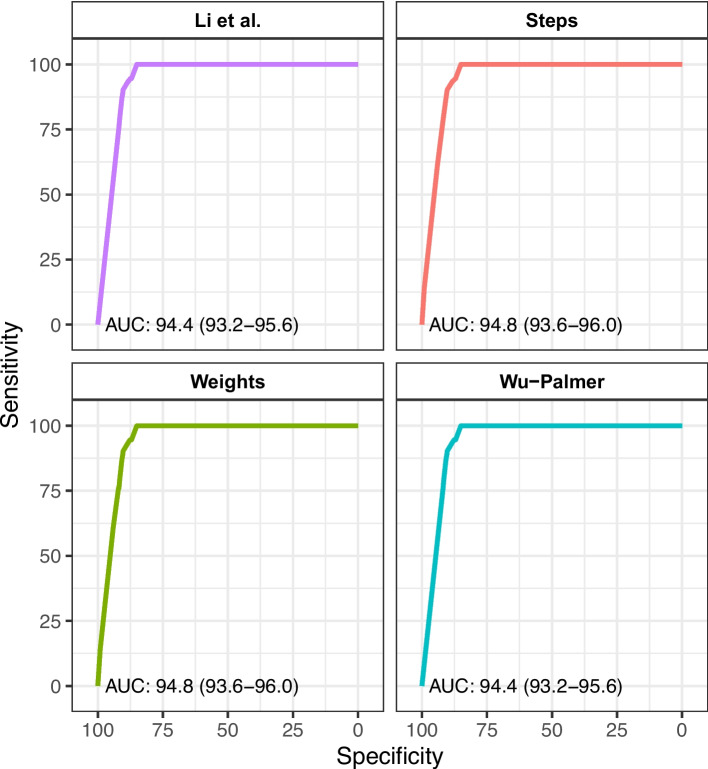
Discriminative performance of the four algorithms to discriminate between similar and discriminant diagnostic labels. AUC, area under the curve. For a detailed description of what sensitivity/specificity is achievable by what cut-off value for the steps parameter, please see Table 2

The ability of the automated metric to discriminate between similar and discrepant diagnoses was high and consistent across algorithms, with an area under the ROC curve (AUC) of 0.94–0.95 (Fig. 1). Post-hoc analyses combining similarity algorithms did not improve discriminative performance; therefore, subsequent analyses were conducted using the simplest step-count algorithm (“steps”).

Table 2 summarizes the discriminative performance of the step-count algorithm at different cutoffs. Using a cutoff of ≥ 2 ICD-10 hierarchical steps to define a discrepancy, all cases manually classified as discrepant were correctly identified by the automated method (100% sensitivity), at the cost of a 15% false-positive rate; 162 non-discrepant cases were incorrectly flagged as discrepant. A cutoff of ≥ 5 steps optimized both sensitivity and specificity, correctly identifying 90% of discrepant and 90% of non-discrepant diagnoses.

**Table 2:** Discriminative power for different cut-offs values for the steps algorithm

Cut-off	Sensitivity	Specificity	FPR	FNR	PPV	NPV
11.00	0.00	1.00	0.00	1.00	-	0.92
10.00	0.00	1.00	0.00	1.00	-	0.92
9.00^a^	0.14	0.99	0.01	0.86	0.57	0.93
8.00	0.61	0.94	0.06	0.39	0.47	0.97
7.00	0.79	0.92	0.08	0.21	0.45	0.98
6.00	0.87	0.91	0.09	0.13	0.44	0.99
5.00^b^	0.90	0.90	0.10	0.10	0.44	0.99
4.00	0.93	0.88	0.12	0.07	0.40	0.99
3.00	0.95	0.87	0.13	0.05	0.38	0.99
2.00^c^	1.00	0.85	0.15	0.00	0.36	1.00
1.00	1.00	0.85	0.15	0.00	0.36	1.00
0.00	1.00	0.00	1.00	0.00	0.08	-

^a^Optimal cut-off to optimize specificity

^b^Optimal cut-off to optimize specificity + sensitivity

^c^Optimal cut-off to optimize sensitivity/Optimal cut-off according to Youden’s Index

*FPR *False positive rate, *FNR *false negative rate, *PPV *positive predictive value, *NPV *negative predictive value

We further analyzed all 162 false-positive cases identified using a cutoff of ≥ 2 steps. Of these, experts classified 88 cases (54%) as increased diagnostic precision and 60 cases (37%) as complications, i.e. new diagnoses that were not foreseeable at the time of ED discharge. Only 14 cases (9%) represented medical or verbatim identical diagnoses that were incorrectly flagged as discrepant, resulting in a true negative predictive value (NPV) of 91%.

## Discussion

In this study, we validated a previously described automated method [18] to identify discrepancies between ED discharge and follow-up diagnoses, using data from 1204 patients enrolled in the DDxBRO trial across four different Swiss emergency departments.

When compared with manual expert physician assessment, the automated classification demonstrated a high discriminatory ability (AUC ≈ 0.95) to flag diagnostic discrepancies in EM using routinely available ICD-10 codes, independent of the specific algorithm used to calculate distances between initial and later diagnoses. This approach shows strong potential as a screening method to identify cases for targeted chart review, thereby supporting the detection of potential diagnostic errors in both research and quality improvement initiatives. Importantly, this method is not intended to replace expert review or to classify harm directly, but rather to make the process of identifying cases for review more efficient and scalable. Also, the fact that the automated classification calculates the distance between two given diagnoses in the ICD taxonomy allows the user of this approach to adjust the cut-off value for when to classify diagnoses as discrepant to their specific needs. In quality assurance projects, where a high sensitivity for discrepancy is warranted, more than 2 steps difference would result at a 100% sensitivity for clinically relevant discrepant diagnoses, at an 85% specificity. In research projects eager to better balance sensitivity and specificity for clinically relevant discrepancies, 5 steps may be a better cut off, resulting in a sensitivity and specificity of 90% each. Table 2 provides the user of the automated classification approach with an overview of what cut off to use depending on their specific needs.

The reference test used in this study was first described in 2016 [20] and was first used in a study that compared emergency department admission diagnoses to hospital discharge diagnoses [17]. Since then, several studies have used this method to manually identify diagnostic labeling errors [21–29]. However, manual classification is labor-intensive and limits its use to smaller datasets. The automated approach validated here offers a more scalable solution, reducing manual review to only those cases automatically flagged as potentially discrepant. In this study, the screening method would have reduced 1,204 diagnosis pairs to 263 for expert review, of which 38.4% were confirmed as discrepant. This demonstrates that the approach can substantially improve efficiency and is well suited for both research and routine quality improvement initiatives. It should however be noted that the approach presented here requires some form of linkage from initial to more definitive diagnosis. In the context of our study, this linkage was created by setting an (arbitrary) time frame from ED visit to follow up diagnosis of no more than 14 days. Likely, a shorter time frame would identify less and a longer time frame more discrepancies, inducing the risk of reduced sensitivity for error or reduced specificity, respectively. Furthermore, it should be noted that rater agreement in manually assessing cases for diagnostic error is notoriously low in the literature, with kappa ranging from 0.00 to 0.04 [30]. The agreement between raters in our study was about ten times higher (kappa 0.4). However, this value is still considered far from perfect.

### Limitations

This study has several limitations. First, not all diagnostic discrepancies represent true errors or harm. Many reflect evolving disease presentations, differences in documentation, or coding variability. The tool should therefore be considered strictly as a screening trigger rather than a definitive classifier of diagnostic error. Automated identification of diagnostic discrepancies can help prioritize cases for resource-intensive chart reviews, such as those performed with the Safer Dx instrument [31]. While labeling discrepancies provide a measurable signal of diagnostic quality, some experts argue that identifying missed diagnostic opportunities offers greater value, as it directly informs education, system design, and process improvement. However, detecting such opportunities requires extensive expert chart review, whereas labeling discrepancies are simpler to measure and scale.

In a sub-analysis of the DDxBRO trial, we demonstrated that patients with a positive diagnostic quality risk score (a composite measure including diagnostic label discrepancy, revisit, unexpected medical care, and death) were 13.5 times more likely to have a missed diagnostic opportunity than those without a negative score (54% vs. 4%) [19]. This suggests that screening for diagnostic discrepancies, followed by targeted chart review, can be an efficient strategy to optimize reviewer time and focus expert attention where it is most needed.

Second, ICD coding itself introduces limitations. Coding inherently reduces diagnostic nuance compared to free-text documentation. A patient’s clinical presentation is often described more precisely in narrative notes than in a single code. However, ICD codes are widely available in many health systems, routinely generated for billing, and sufficient for many quality and safety applications. The ICD-10 codes used in this study were collected by trained physicians explicitly for research purposes. It remains to be determined whether performance would be similar when using routine billing codes, which may be less precise.

Third, we validated our approach in a dataset from the DDxBRO trial [19]. This trial has intentionally recruited patients considered “difficult to diagnose”. Indeed, the overall misdiagnosis rate of 16% reported in the DDxBRO trial is slightly higher than previous estimates of diagnostic error in emergency medicine, ranging at around 10% [9, 17, 24]. As a consequence, the approach presented here may not be generalizable to all ED patients. At least, its applicability should be tested in a further study.

## Conclusion

This study demonstrates that an automated trigger system for detecting potential diagnostic labeling discrepancies performs with high accuracy compared to expert physician review. The freely available software evaluated here shows promise as a practical tool to support diagnostic error research and quality improvement in emergency medicine by identifying cases that merit detailed chart review. Future studies should examine its performance with routinely collected ICD-10 billing codes and across diverse clinical settings to confirm its generalizability and utility in routine quality monitoring.

## Supplementary Information

Additional file 1.

## Data Availability

The datasets used and/or analysed during the current study are available from the corresponding author on reasonable request.
