# Supplementary material for: Identification of diagnostic discrepancies as a quality assurance measure in emergency medicine – a validation study
Source: Scand J Trauma Resusc Emerg Med. 2026 Feb 11;34:56. doi: 10.1186/s13049-026-01572-x (PMC12998101; doi:10.1186/s13049-026-01572-x)
Supplement: Supplementary file 1 — Additional file 1. [file 13049_2026_1572_MOESM1_ESM.docx]

# Supplementary Methods

## Rating of diagnostic discrepancy: reference standard

Changes in label of the primary diagnosis from ED discharge to time of follow-up were rated by two blinded investigators not involved in the procedure of the clinical trial according to predefined rules and scheme. Both investigators were experienced ED physicians. Both investigators rated 40 cases each, which were subsequently discussed with a third investigator with extensive experience in emergency medicine and research on diagnostic labeling errors to assess questions regarding the rating process and the classification rules and scheme. Subsequently, another 100 cases were rated by both investigators separately to calculate interrater variability, resulting in fair to moderate agreement (Cohen’s kappa = 0·4), meeting the predefined threshold of 0·4 or above. Differences in ratings for the first 140 cases were resolved by discussion between the two raters. For the remaining cases, each case was assessed by one of the two raters alone.

### Classification scheme for diagnostic discrepancies

Changes in diagnosis at follow-up were rated according to a predefined classification scheme published in Hautz WE, Kämmer JE, Hautz SC et al. Diagnostic error increases mortality and length of hospital stay in patients presenting through the emergency room. *Scand J Trauma Resusc Emerg Med.* 2019;**27**:54. <https://doi.org/10.1186/s13049-019-0629-z>, see next page


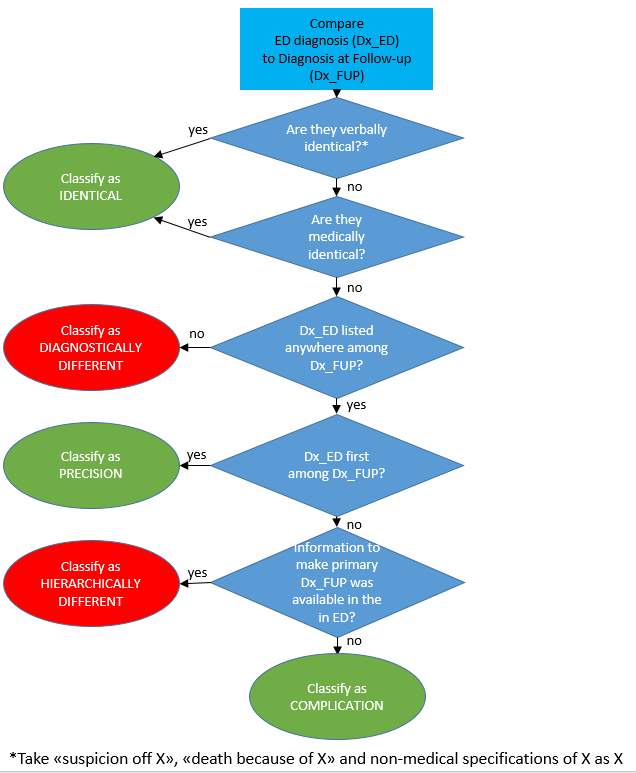

Note: ED, emergency department; FUP, follow-up

## Handling of diagnoses from ICD chapter R

Diagnoses from ICD chapter R “(Symptoms, Signs and abnormal findings”) are frequent in Emergency Medicine (and often also adequate, such as R 10.4 ”abdominal pain, not classified elsewhere”). Quite frequently, it is impossible to find the cause of such pain with reasonable diagnostic effort – and quite often, the pain resolves within days. As a result, R diagnoses are common and often defensible in emergency medicine. There are 261 R-diagnoses made in the ED in the DDx-BRO data set (21.7%).

In expert review, such an R diagnosis would be compared to the follow up diagnosis manually, see section 1.1. If, for example R 10.4 changed to R 85.0 (“benign peritonitis”), experts would likely classify these codes as not being of relevant difference. If, however, the follow up diagnosis was acute Cholecystitis (K81.0), likely experts would classify this as a relevant diagnostic discrepancy. The index test in this study however would simply count the steps required from one to the other diagnosis and (with a cutoff at 2 steps) would classify both diagnoses as discrepant. That is exactly why manual review of discrepant cases would be required after automated analysis.
